# Mechanisms of natural compounds in treating inhalation anesthesia-induced cognitive dysfunction

**DOI:** 10.3389/fphar.2026.1853638

**Published:** 2026-07-01

**Authors:** Zhaobing Han, Wei Jiang, Yue Zhao, Yannan Geng, Yuanjun Qu, Qiuyue Chen, Xiwen Geng, Guimao Cao

**Affiliations:** 1 Second School of Clinical Medicine, Shandong University of Traditional Chinese Medicine, Ji’nan, China; 2 Experimental Center, Shandong University of Traditional Chinese Medicine, Ji’nan, China; 3 First School of Clinical Medicine, Shandong University of Traditional Chinese Medicine, Ji’nan, China; 4 Department of Pharmacy, Affiliated Hospital of Shandong University of Chinese Medicine, Ji’nan, China; 5 Key Laboratory of Traditional Chinese Medicine Classical Theory, Ministry of Education, Shandong University of Traditional Chinese Medicine, Ji’nan, China; 6 The Affiliated Hospital, Shandong University of Traditional Chinese Medicine, Ji’nan, China; 7 Department of Anesthesiology, Kashi Prefecture Hospital of Traditional Chinese Medicine, Kashi, Xinjiang, China

**Keywords:** cognitive dysfunction, inhalation anesthetics, natural compounds, neuroinflammation, neuronal apoptosis, oxidative stress

## Abstract

Although some inhalation anesthetics have been widely used and improved surgical safety, their potential to cause cognitive decline and neurotoxicity has raised widespread concern. The pathological mechanisms of cognitive dysfunction induced by anesthesia are complex, involving multiple factors such as neuroinflammation, oxidative stress, neuronal apoptosis, microglial polarization, neurotransmitter imbalance, as well as miRNA and epigenetic regulation. However, current treatment options are limited, and there is a lack of targeted and safely effective intervention measures. Natural compounds demonstrated significant potential in the prevention and treatment of cognitive dysfunction induced by anesthesia due to their multi-target regulatory properties and good safety profiles. This review systematically explores the potential role of natural compounds in alleviating cognitive dysfunction induced by inhalational anesthetics. A total of 16 natural compounds were evaluated, including flavonoids; Saponins; Phenols/Polyphenols; Terpenes, and other compounds. The main mechanisms of action identified by the research include anti-inflammatory effects, antioxidant effects, anti-apoptotic effects, regulation of microglial polarization, and epigenetic regulation. Furthermore, the current challenges and future research directions in this field were clarified.

## 1 Introduction

Anesthesia-induced cognitive dysfunction is a family of conditions typified by the deterioration of cognitive processes, including memory, attention and psychomotor abilities as well as information processing skills, which can be particularly caused by anesthesia (Li et al., 2022b; Zheng et al., 2024). It may even cause irreversible neurologic damage, impairing daily functions and social adaptability clinically. Sevoflurane and isoflurane are examples of inhalation anesthetic which are commonly used today because they have a quick onset and can be comfortably controlled (Zhang et al., 2023). Nevertheless, prolonged or repeated exposure can cause neuropathological alterations and cognitive dysfunction (Wang et al., 2021).

Epidemiological statistics show that as the percentage of the ageing population increases throughout the globe, the elderly population will escalate to about two billion in 2050, with an estimated 30% of the aged patients needing general anesthesia (Zhou et al., 2021). This indicates that there will be more elderly people receiving anesthesia in the future, posing a major challenge in preventing and treating anesthesia-induced cognitive dysfunction (Zheng et al., 2024). These conditions not only have a significant impact on patients’ hospital stay, morbidity and mortality, but also result in impaired recovery, higher complications, and even loss of independence in living abilities, imposing a huge psychological and economic burden on patients and their families in addition to causing increased hospitalizations and higher healthcare expenditures. However, current clinical treatment methods still have limitations: existing guidelines recommend cognitive function assessments prior to surgery for patients aged over 65, caution regarding the use of drugs that may increase the risk of postoperative delirium, maintenance of hemodynamic stability during surgery, and the use of electroencephalography monitoring to guide anesthetic depth, yet no specific anesthetic drugs have been recommended for the prevention of cognitive dysfunction (Belrose and Noppens, 2019). Besides, there is a lack of effectively specific treatment methods for anesthesia-induced neurotoxicity and cognitive disorders, primarily relying on supportive treatments, while some anti-inflammatory drugs exhibit non-tissue specificity and significant side effects, with clinical safety remaining uncertain (Belrose and Noppens, 2019; Ma et al., 2024b; Zhang et al., 2025a).

**FIGURE 1:**
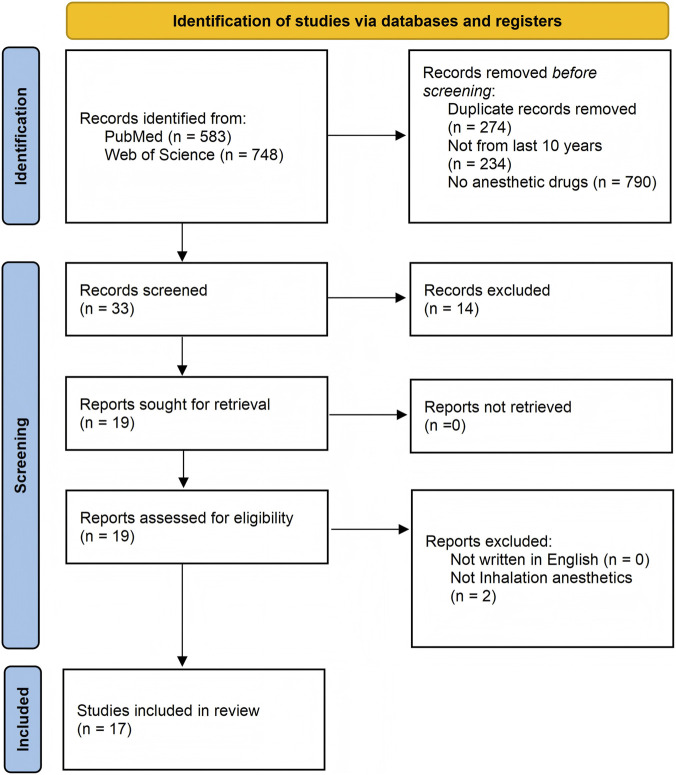
Flow diagram illustrating the literature search methodology of this review.

Some natural compounds have shown significant research value as potential therapeutic agents due to their multi-target regulatory properties, low toxicity, and relatively good safety profile (Aktary et al., 2025). The mechanisms involved in the anesthesia-induced cognitive dysfunction include neuroinflammation (release of pro-inflammatory factors such as tumor necrosis factor alpha (TNF-α), interleukin-1beta (IL-1β), and interleukin-6 (IL-6), neuronal apoptosis (activation of caspase-3 and imbalance of Bcl-2 family proteins), and oxidative stress (Li et al., 2022b; Luo et al., 2025). Research has shown that pharmacological properties of natural compounds are anti-inflammatory, antioxidant, and neuronal signaling pathways modulation (Chopra and Dhingra, 2021). These characteristics make them potentially useful in neuroprotection and treatment of anesthesia-induced cognitive dysfunction. The mechanisms of action mostly entail the intervention of pathological mechanisms like neuroinflammation, oxidative stress, neuronal apoptosis, and neural plasticity damage caused by anesthetics (Luo et al., 2025). Not only do these studies unveil the neuroprotective capability of the natural compounds (Kim et al., 2025; Li et al., 2025a; Ogunro, 2025; Yuan et al., 2025; Zhang et al., 2025b; Ji et al., 2026), but they also present empirical evidence on developing novel drugs to address cognitive dysfunction. The purpose of this review is to outline the current advancements in natural compound mechanisms in cognitive dysfunction resulting from anesthesia and the gaps, to provide future directions of development and therefore to provide theoretical reasoning to support the treatment methods of cognitive dysfunction caused by anesthesia using natural compounds and to facilitate basic and clinical research in this field. Figure 1 illustrates the mechanisms by which natural compounds and anesthesia lead to cognitive impairment.

## 2 Methodology for the literature search and included studies

### 2.1 Focal question

The central question guiding the development of this review was: “What are the mechanisms by which medicinal plants and natural plant chemicals regulate anesthesia-induced cognitive dysfunction?”

### 2.2 Language

This review exclusively incorporated studies that were written in English.

### 2.3 Databases

PubMed, Web of Science databases were searched. The mesh terms employed in this study were “natural phytochemicals”, “synthesized phytochemicals”, “medicinal plants”, and “botanical medicine” in combination with “Cognitive dysfunction”, “Anesthetic drugs”, “Inhalation anesthesia”, “Sevoflurane”, and “Isoflurane”. We did not contact investigators, nor did we attempt to find unpublished data.

### 2.4 Study selection

This review selected all literature related to the research topic from the past 10 years for analysis, considering only full-text versions. Only full texts were considered.

### 2.5 Data extraction

**FIGURE 2:**
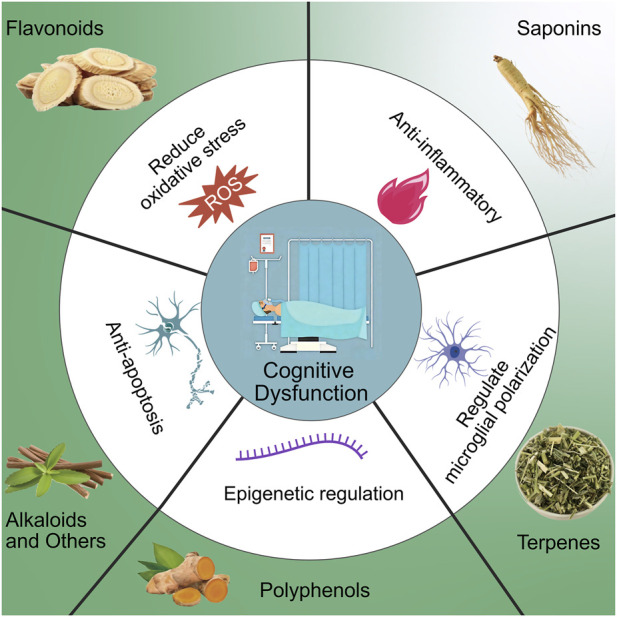
Types and mechanisms of action of natural compounds.

The studies investigating the utilization of medicinal plants and natural phytochemicals for regulating anesthesia-induced cognitive dysfunction are listed in Tables 1, 2, respectively. Figure 2 illustrates the literature search.

**TABLE 1:** Research information of natural compounds in treating inhalation anesthesia-induced cognitive dysfunction.

Category	Anesthetic	Animal and treatment	Compound	Source	Primary mechanism	Limitations of the study	Main evaluation indicators	References
Flavonoids	Sevoflurane	Rat i.p.	Hispidulin	*Saussurea involucrata* (Kar. and Kir.) Sch.Bip. [Asteraceae]	Specific activation of the AMPK-mTOR pathway induces autophagy	The dose-effect relationship of hispidulin has not been evaluated	Morris water maze: Escape latency, Platform crossing times, Time spent in target quadrant	Huang et al. (2018)
	Isoflurane	Rat i.c.v.	Quercetin	*Hippophae rhamnoides* L. [Elaeagnaceae]	Inhibiting neuroinflammation by regulating the miR-138–5p/LCN2 axis	The administration method of quercetin was not specified, and there is a lack of evaluation for the optimal dosage and safety	Morris water maze: Escape latency, Traveling distance, Residence time in target quadrant, Platform crossing times	Lou et al. (2025)
	Sevoflurane	Mouse i.p.	Luteoline	*Lonicera japonica* Thunb. [Caprifoliaceae]	Inhibition of the NF-κβ/NLRP3 inflammasome pathway	No dose-gradient exploration was conducted for luteoline, and the cumulative toxicity or tolerability of long-term, repeated administration was not evaluated	Morris water maze: Escape latency, Platform crossing times	Wang et al. (2019)
	Sevoflurane	Rat Standard pelleted diet	Baicalein	*Scutellaria baicalensis *Georgi [Lamiaceae]	Regulate multiple signaling pathways such as PI3K/Akt/GSK-3β and JNK/ERK MAPK, with anti-apoptotic effects	No toxicity assessment was conducted for the dose used, and the sevoflurane exposure protocol (3% for a continuous 6 h) was far higher than the concentrations and durations commonly used in clinical practice	Open field test: distance travelled in the field in 10 min; Fear conditioning test: freezing time; Olfactory test: decrease the latency time to find the buried food Morris water maze: Escape latency, Time spent in target quadrant	Wang and Zhou (2018)
	Isoflurane	Rat i.g.	Rutin	*Fagopyrum esculentum* Moench [Polygonaceae]	Inhibit the JNK/p38 MAPK pathway, anti-apoptosis	Toxicity assessment of the dose used was not conducted, and causal validation was not performed using pathway-specific inhibitors or gene knockdown	Morris water maze: Escape latency, Time spent in target quadrant	Li et al. (2017a), Li et al. (2017a)
Saponins	Sevoflurane	Rat i.p.	Chikusetsu saponin IVa	*Panax* *japonicus* C.A.Mey. [Araliaceae]	Block the NLRP3/caspase-1 inflammasome pathway	The dose-response relationship of Chikusetsu saponin IVa was not evaluated; the sevoflurane exposure protocol (4% for 3 h) featured both a high concentration and a long duration	Morris water maze: Escape latency, Traveling distance, Crossing times on the original platform, Residence time in target quadrant; NOR test: the time spent in the new object, the time spent in the familiar objects; Y-maze test: the number of successive (successive spontaneous alternation) SA, the total number of times to explore the maze	Shao et al. (2020)
	Isoflurane	Rat i.p.			Activate the downstream ERK1/2 signaling pathway by upregulating SIRT1 expression	Neither the optimal effective dose has been determined nor the pharmacological correlation between the *in vitro* experiment (25 μg/ml) and the *in vivo* dose has been clarified	Morris water maze: Escape latency, Escape path length, Residence time in target quadrant, Platform crossing times	Fang et al. (2017)
	Isoflurane	Rat i.p.	Ginsenoside Rg1	*Panax ginseng* C. A. Mey. [Araliaceae]	Activate the PI3K/Akt pathway, mediating a combined effect of antioxidant, anti-inflammatory, and anti-apoptotic actions	No dose gradient exploration was conducted, and only the Morris water maze was used in behavioural studies, without being combined with other cognitive tests	Morris water maze: Escape latency, Traveling distance, Mean swim speed, Swimming duration	Zhang et al. (2016)
Phenols and Polyphenols	Isoflurane	Rat i.g.	Paeonol	*Paeonia × suffruticosa* Andrews [Paeoniaceae]	Regulating the JNK/ERK/p38 MAPK pathway and histone acetylation	Use of pathway-specific inhibitors or gene knockdown for causal validation was not carried out	Morris water maze: Escape latency; Fear conditioning test: freezing time; Open field test: distance travelled in the field in 5 min	Jin et al. (2020)
	Sevoflurane	Mouse i.p.	Resveratrol	*Veratrum nigrum* L. [Melanthiaceae]	Activate SIRT1, deacetylate and inhibit NF-kβ	A full dose-response relationship was not explored, and the behavioural assessment was limited to the Morris water maze, employing a relatively singular evaluation methodology	Open field test: distance travelled in the field in 5 min, the average speed; Morris water maze: Escape latency, Traveling distance, Residence time in target quadrant, Crossing time on the original platform	Tang et al. (2021)
	Isoflurane	Rat i.p.	Curcumin	*Curcuma longa* L. [Zingiberaceae]	Downregulate the expression of miR-181a-5p to inhibit neuroinflammation	No separate control group for Curcumin was established, therefore it is not possible to rule out the effect of Curcumin itself on cognitive function	Morris water maze: Escape latency, Traveling distance, Residence time in target quadrant, Crossing time on the original platform	Liu et al. (2021)
	Isoflurane	Mouse i.p.	Green tea polyphenols	*Camellia sinensis* (L.) Kuntze [Theaceae]	Increase p-CaMKII, p-CREB, and BDNF to protect synaptic function	Fewer samples for each group were used in the SOD and proteins levels examinations, than used in fear conditioning test and novel objection recognition	Fear conditioning test: The percentage of freezing time; NOR test: discrimination index, novel object preference ratio	Song et al. (2019)
Terpenes	Sevoflurane	Mouse i.p.	Morroniside	*Cornus officinalis* Siebold & Zucc. [Cornaceae]	Inhibit the TLR4/NF-κβ pathway and promote the polarization of microglia to the M2 type	Sample size is small and behavioural assessment only uses the Morris water maze, with a single assessment method	Morris water maze: Escape latency, mean path length, Residence time in target quadrant, Crossing time on the original platform	Chen et al. (2025)
​	Isoflurane	Rat i.p.	Picroside II	*Neopicrorhiza scrophulariiflora (Pennell)* D. Y. Hong [Plantaginaceae]	By downregulating the expression of miR-195, it subsequently inhibits the inflammatory response and exerts neuroprotective effects	No other neuronal markers were detected in the hippocampal tissue, and behavioural assessment was limited solely to the Morris water maze, representing a single evaluation method	Morris water maze: Escape latency, Time spent in target quadrant	Li et al. (2022a)
	Sevoflurane	Rat Tail vein injection	Gynosaponin	*Gynostemma pentaphyllum* (Thunb.) Makino [Cucurbitaceae]	Activate the PI3K/Akt/mTOR pathway to inhibit neuronal apoptosis	No dose-gradient exploration was conducted, and behavioural assessment utilised only the Morris water maze, resulting in a single evaluation method	Morris water maze: Escape latency, Platform crossing times, Time spent in target quadrant; Open field test: distance travelled in center area, Time in central area	Lin et al. (2024)
Alkaloids and Others	Isoflurane	Rat i.p.	Eleutheroside E	*Eleutherococcus senticosus* (Rupr&Maxim) Maxim. [Araliaceae]	Regulating the α7-nAChR–NMDAR pathway to improve cholinergic neurotransmission	No dose-gradient exploration was conducted, and causal verification was not performed using specific agonists/antagonists of α7-nAChR	Morris water maze: Escape latency, swim speed and time in the target quadrant; NOR test: the time spent in the new object, the time spent in the familiar objects, Total distance traveled	Lu and Xiao-Qing (2019)
	Sevoflurane	Mouse i.g.	Tanshinone IIA	*Salvia miltiorrhiza* Bunge [Lamiaceae]	Inhibit neuronal cell apoptosis, protect synaptic structure, anti-apoptotic and neuroprotective effects	No dose gradient exploration was conducted, and the behavioural assessment only utilised fear conditioning text, resulting in a single evaluation method	Open field test: distance travelled in the field in 5 min, Time spent in the center; Fear conditioning test: Freezing time to context, Freezing time to tone	Xia et al. (2017)

Akt, Protein Kinase; AMPK, AMP Activated Protein Kinases; Bad, Bcl2-Associated Agonist of Cell Death Protein; Bax, Bcl2-Associated X Protein; c-JUN, Jun Proto-Oncogene; BCL-2, B-cell lymphoma 2; BCL-XL, B-cell lymphoma-extra large; BDNF, Brain-Derived Neurotrophic Factor; ERK1/2, Extracellular-Signal-Regulated Kinase 1/2; GSK-3β, Glycogen Synthasekinase-3β; IL-1β, Interleukin-1beta; IL-6, Interleukin-6; JNK, c-Jun N-terminal Kinase; LCN2, Lipocalin 2; MAPK, Mitogen-Activated Protein Kinases; mTOR, Mammalian Target of Rapamycin; MEK, Mitogen-Activated Protein Kinase Kinase; NF-кβ, Nuclear Factor Kappa β; NLRP3, NOD-Like Receptor 3; P-CaMKII, Phospho-Thr286-Calcium-Calmodulin Dependent Protein Kinase II; p-CREB, Phospho-Ser133-CAMP-Dependent Response Element Binding Protein; PDK1, Phosphoinositide-dependent kinase-1 PI3K, Phosphoinositide 3-Kinase; P38, P38 mitogen-activated protein kinase; Raf, Rapidly Accelerated Fibrosarcoma Kinase; RAS-GTP, Ras-Specific GTPase-Activating Proteins; Rheb, Ras Homolog Enriched in Brain; SIRT, A Homologous Family of Regulatory Enzymes Containing 3; SOD, Superoxide Dismutase; TLR4, Toll-Like Receptor 4; TNF-α, Tumor Necrosis Factor Alpha; TSC1/2, Tuberous Sclerosis Complex 1/2; α7-nAChR, Alpha7-Nicotinic Acetylcholine Receptors.

**TABLE 2:** Key pharmacological parameters of the included studies.

Category	Anesthetic	Animal and treatment	Compound	Dose/Concentration range	Duration	Positive control	Negative control	References
Flavonoids	Sevoflurane	Rat i.p.	Hispidulin	40 mg/kg	Single dose at12 h prior to anesthesia	Yes	Yes	Huang et al. (2018)
	Isoflurane	Rat i.c.v.	Quercetin	5 mg/kg	Not specified	Yes	Yes	Lou et al. (2025)
	Sevoflurane	Mouse i.p.	Luteoline	30, 60 mg/kg	Single dose at 30 min prior to anesthesia	No	Yes	Wang et al. (2019)
	Sevoflurane	Rat Standard pelleted diet	Baicalein	25, 50, 100 mg/kg	Daily from P3 to P21	No	Yes	Wang and Zhou (2018)
	Isoflurane	Rat i.g.	Rutin	10, 20, 40 mg/kg	Daily from P1 to P15	No	Yes	Li et al. (2017a)
Saponins	Sevoflurane	Rat i.p.	Chikusetsu saponin IVa	30 mg/kg	Single dose at 12 h prior to anesthesia	Yes	Yes	Shao et al. (2020)
	Isoflurane	Rat i.p.		15–60 mg/kg	Single dose at 30 min prior to anesthesia	Yes	Yes	Fang et al. (2017)
	Isoflurane	Rat i.p.	Ginsenoside Rg1	20 mg/kg	Daily for 7 consecutive days	No	Yes	Zhang et al. (2016)
Phenols and Polyphenols	Isoflurane	Rat i.g.	Paeonol	50, 100, 150 mg/kg	Daily from P2 to P21	No	Yes	Jin et al. (2020)
	Sevoflurane	Mouse i.p.	Resveratrol	100 mg/kg	Daily for 6 consecutive days	No	Yes	Tang et al. (2021)
	Isoflurane	Rat i.p.	Curcumin	200 mg/kg	Not specified	Yes	Yes	Liu et al. (2021)
	Isoflurane	Mouse i.p.	Green tea polyphenols	25 mg/kg×7 d 75 mg/kg	Multi-dose: daily for 7 days; Single-dose: once on day 7	No	Yes	Song et al. (2019)
Terpenes	Sevoflurane	Mouse i.p.	Morroniside	30, 60, 100 mg/kg	Every 3 days for 4 weeks	Yes	Yes	Chen et al. (2025)
	Isoflurane	Rat i.p.	Picroside II	10–30 mg/kg	Daily for 5 days	No	Yes	Li et al. (2022a)
	Sevoflurane	Rat Tail vein injection	Gynosaponin	25, 100 mg/kg	Single dose 1 h prior to anesthesia	Yes	Yes	Lin et al. (2024)
Alkaloids and Others	Isoflurane	Rat i.p.	Eleutheroside E	50 mg/kg	Single dose 1 h prior to anesthesia	No	Yes	Lu and Xiao-Qing (2019)
	Sevoflurane	Mouse i.g.	Tanshinone IIA	10, 20 mg/kg	Daily for 3 days	No	Yes	Xia et al. (2017)

## 3 Overview of the pathological mechanisms of anesthesia-induced cognitive dysfunction

### 3.1 The role of neuroinflammation in cognitive dysfunction caused by anesthesia

Neuroinflammation is an immune response mechanism generated by the central nervous system to respond to injury or stress. Moderate neuroinflammation is protective, but when it becomes excessive, neurons may be damaged and cognitive dysfunction occur. It has been established that neuroinflammation is one of the most important pathological processes that occur in cognitive dysfunction as a result of anesthesia, where anesthetic agents are capable of stimulating neuroinflammatory processes, resulting in neuronal injury in brain regions involved in cognition, such as the hippocampus, leading to cognitive dysfunction (Xu et al., 2022; Liu et al., 2023). Isoflurane and sevoflurane are examples of anesthetic agents that can trigger neuroinflammatory pathways to a significant degree, as evidenced by the excessive release of pro-inflammatory cytokines (Alhayyan et al., 2020; Zhou et al., 2022; Yang et al., 2023). Exposure to isoflurane has been found to cause neuronal damage and cognitive dysfunction in the hippocampus of rats, and increased expression of the pro-inflammatory cytokines IL-1β, IL-6, and TNF-α(Li et al., 2022a; Huang et al., 2025). The levels of the pro-inflammatory factors mentioned above are also significantly increased in the hippocampal tissue of the mice after inhalation of sevoflurane (Chen et al., 2025). Moreover, sevoflurane is capable of causing the increase in the expression of pro-inflammatory cytokines in the hippocampal area of rats, which results in neuroinflammation, the occurrence of neuronal apoptosis, and the overproduction of reactive oxygen species (ROS), which leads to the impairment of cognitive functions (Shao et al., 2020; Zuccoli et al., 2024; Deng et al., 2025; Yang et al., 2025). The release of these pro-inflammatory cytokines can directly damage neuronal structure and function, disrupt synaptic plasticity, and thus affect cognitive processes such as learning and memory (Baune et al., 2008; McAfoose and Baune, 2009; Gimsa et al., 2012; Miller et al., 2013; Silva et al., 2022).

### 3.2 Oxidative stress, neuronal apoptosis, and its associated signaling pathway

Oxidative stress refers to the pathological process in which an imbalance between oxidation and antioxidation occurs due to excessive production of ROS or weakened function of the antioxidant defense system in the body when subjected to various harmful stimuli, resulting in damage to cell structure and function. Anesthetics can induce oxidative stress, leading to oxidative damage in nerve cells, which participates in the onset of cognitive dysfunction. The molecular mechanisms mainly involve the activation of apoptotic pathways by oxidative stress and the regulation of multiple signaling transduction pathways (Jiang et al., 2022; Zhou et al., 2023; Lu et al., 2024b).

Neuronal apoptosis is a significant pathological outcome in oxidative damage caused by anesthesia. There are several pathways that anesthetics can induce apoptosis of nerve cells, which contributes to the development of cognitive dysfunction. Using isoflurane as an example, exposure to it greatly enhances apoptosis of nerve cells in the hippocampal area of experimental animals (Wang et al., 2009; Lin and Zuo, 2011; Hu et al., 2024; Xu et al., 2024). In a neonatal rat model, isoflurane treatment significantly enhances the count of TUNEL-positive cells in the cornu ammonis one area (CA1), cornu ammonis three area (CA3), and dentate gyrus regions of the hippocampus and displays abnormal expression of proteins that promote apoptosis (Li et al., 2017a), including higher levels of active caspase-3, pro-apoptotic proteins Bcl2-Associated X Protein (Bax) and Bcl2-Associated Agonist Of Cell Death Protein (Bad), while the expression levels of anti-apoptotic proteins Bcl-2, Bcl-xL, and phosphorylated Bad significantly decrease. This apoptotic process is closely related to the activation of the mitogen-activated protein kinases (MAPK) signaling pathway, specifically manifested by increased phosphorylation levels of c-Jun N-terminal kinase (JNK), extracellular-signal-regulated kinase 1/2 (ERK1/2), and p38 (Li et al., 2017a), where the JNK signaling pathway can mediate nerve cell apoptosis by regulating the activation of Bcl-2 family proteins and c-Jun (Wagner et al., 1999; Guan et al., 2005; Han et al., 2008; Li et al., 2013). Moreover, studies on developing rats have also confirmed that isoflurane exposure leads to an increase in cleaved caspase-3 expression in the hippocampal region and a higher number of TUNEL-positive cells, further suggesting that isoflurane-mediated neurotoxicity is accompanied by a significant apoptotic process (Li et al., 2017b).

In addition, exposure to the inhalational anesthetic sevoflurane can lead to a significant increase in neuronal apoptosis in naive mice and rats (Zheng et al., 2013; Zhu et al., 2024), typically characterized by an increase in TUNEL-positive cell counts in the hippocampus and elevated expression of activated caspase-3, suggesting that oxidative stress is closely related to the activation of the apoptotic pathway (Dong et al., 2009; Lu et al., 2010). As the main site of cellular oxidative metabolism, mitochondrial dysfunction plays a key role in oxidative stress-mediated neuronal injury: oxidative stress caused by sevoflurane can cause disruption in the expression of apoptotic-related proteins, with upregulation of pro-apoptotic proteins Bax and Bad and downregulation of anti-apoptotic proteins Bcl-2 and Bcl-xL, indicating that the mitochondrial pathway is involved in the apoptosis process triggered by oxidative stress (Wang and Zhou, 2018; Cheng et al., 2024).

The above mechanisms participate in anesthetic-induced neurotoxicity by regulating multiple signaling pathways. In neuronal injury caused by sevoflurane, key relevant signaling pathways include the phosphoinositide 3-kinase (PI3K)/protein kinase B (Akt)/glycogen synthase kinase-3β (GSK-3β) pathway and the JNK/ERK pathways within the MAPK family. Sevoflurane can inhibit the activity of the PI3K/Akt signaling pathway, decreasing the levels of Akt, GSK-3β, and their phosphorylation, which weakens the cell survival signal; simultaneously, it activates the JNK pathway (elevating the phosphorylation levels of JNK and its downstream target c-Jun) and inhibits the activity of ERK1/2, collectively creating a signaling environment that promotes apoptosis (Wang et al., 2013, 2016b; 2016a; Bi et al., 2018; Wang and Zhou, 2018; Gu et al., 2025; Li et al., 2025b, Li et al., 2025c). In addition, isoflurane anesthesia can lead to decreased activity of superoxide dismutase (SOD) in the hippocampus of mice, implying a weakened antioxidant response, and downregulation of the important signaling molecules phospho-thr286-calcium-calmodulin dependent protein kinase II (p-CaMKII), phospho-ser133-cAMP-dependent response element binding protein (p-CREB) and the neurotrophic factor brain-derived neurotrophic factor (BDNF) that regulate long-term potentiation in the hippocampus. These molecule malfunctions might also contribute to the worsening of cognitive dysfunction through oxidative stress (Song et al., 2019). Another important signaling pathway is the SIRT1/ERK1/2 pathway. Studies have found that isoflurane considerably inhibits the expression of SIRT1 and the level of phosphorylated ERK1/2 (p-ERK1/2) in the hippocampus of neonatal rats. In contrast, the plant extract, Chikusetsu saponin Iva, can activate the SIRT1/ERK1/2 pathway, reducing neuronal apoptosis and lactate dehydrogenase release, while reversing the decrease in the expression of the postsynaptic density protein (PSD-95) caused by isoflurane. These suggest that this pathway exerts a critical role in regulating oxidative stress-related synaptic dysfunction (Fang et al., 2017).

### 3.3 Microglial polarization and neuroimmune regulatory mechanisms

Activated microglia typically exist in two polarized states: the classical M1 pro-inflammatory phenotype and the M2 anti-inflammatory phenotype. However, recent research indicates that the M1/M2 dichotomy is an overly simplified conceptual framework, representing only two extreme activation states. The states of microglia *in vivo* are far more complex than *in vitro*, potentially encompassing multiple distinct yet overlapping functional phenotypes. For example, a growing body of evidence suggests that M2 phenotype subgroups such as M2a, M2b, M2c, and Mox possess unique physiological characteristics and distinct biological functions (Mosser and Edwards, 2008; David and Kroner, 2011; Moore et al., 2013). Nevertheless, the broad M1 and M2 classification has persisted as a useful concept, aiding our deeper understanding of the functional states of microglia during the progression of injury. The imbalance of microglial polarization, characterized by the overactivation of M1 microglia and the dysfunction of M2 microglia, can promote the development of neuroinflammatory damage. Therefore, the polarization of microglia exerts a critical role in the pathological mechanism of anesthetic-induced cognitive dysfunction (Qiu et al., 2020). Anesthetics can influence the intensity and direction of neuroinflammatory responses by regulating the polarization balance of microglia and downstream related signaling pathways, thereby altering cognitive function.

The occurrence of cognitive dysfunction caused by anesthesia is closely related to the excessive activation of microglia and pro-inflammatory phenotype polarization. Systemic inflammation triggered by surgery or anesthesia can activate microglia, promoting their polarization towards the M1-type cells (Cheng et al., 2022; Cheng et al., 2025; Yang et al., 2022; Wei et al., 2023) and releasing pro-inflammatory factors such as IL-1β, TNF-α, and IL-6. These inflammatory factors not only directly impair neuronal function but can also further activate microglia through feedback mechanisms, forming a vicious cycle of “inflammation-microglial activation,” which exacerbates the neuroinflammatory cascade response. Inhalation anesthetics such as sevoflurane can significantly activate microglia and promote their polarization towards the M1-type cells (Sun et al., 2022; Zeng and Zhang, 2023; Jin et al., 2024; Wang et al., 2025), leading to elevated levels of pro-inflammatory factors in the hippocampus, disrupting homeostasis of neurons and the integrity of the blood-brain barrier (Zhao et al., 2023; Ma et al., 2024a; Yu et al., 2024). In contrast, some anesthetic agents have been shown to have neuroprotective effects by inhibiting M1-type cells or promoting M2-type cells. An example is dexmedetomidine, which suppresses the release of pro-inflammatory factors, including TNF-α and IL-1β by inhibiting the toll-like receptor 4 (TLR4)/nuclear factor kappa B (NF-κβ) and ERK/MAPK signaling pathways, while also suppressing M1-type microglial polarization and promoting M2-type microglial polarization, thereby alleviating neuroinflammation and cognitive dysfunction (Zhou et al., 2019; Qiu et al., 2020). Conversely, the intravenous anesthetic etomidate presents pro-inflammatory effects, inducing microglial activation and the formation of neurotoxic A1 astrocytes, which further exacerbate oxidative stress and neuroinflammation, promoting the occurrence of cognitive dysfunction (Li et al., 2020; Xiuwen, 2023; Lu et al., 2024a).

### 3.4 miRNA and epigenetic regulation mechanisms

In the mechanism of cognitive dysfunction induced by anesthetics, changes in epigenetic regulation and variations in microRNA expression play important roles. Research has revealed that anesthetic drugs and surgery have the ability to cause changes in epigenetic modifications, including DNA methylation, histone modifications, and non-coding RNA regulation. These changes are involved in regulating the expression of genes related to neuroinflammation and oxidative stress, thereby significantly affecting cognitive function (Ju et al., 2016; Wu et al., 2016; Chen et al., 2017; Wu and Zhao, 2018; Cabrera et al., 2021; Fan et al., 2021; Chai et al., 2022; Rump and Adamzik, 2022). Among them, miRNA, one of the key elements of non-coding RNA, plays a decisive role in the pathogenesis of cognitive dysfunction after anesthetics by regulating the levels of inflammatory factors and neurotransmitters.

In particular, a particular miRNA affects cognitive functioning by modulating signaling pathways associated with inflammation. Indicatively, it has been determined that miR-129 has the power to counter neuroinflammation through the control of the TLR4/NF-κB signaling pathway, thereby reducing anesthetic-induced cognitive dysfunction. Anesthetic adjuncts, like dexmedetomidine, can inhibit the inflammatory pathway through the upregulation of miR-129 expression and further indicate that miRNA-mediated signaling pathways are potential regulatory targets (Wei et al., 2021). In addition, animal studies have indicated that isoflurane anesthesia causes cognitive dysfunction in rats, it causes a dramatic downregulation of miR-138–5p in hippocampal tissue; downregulation of miR-138–5p expression causes cognitive dysfunction, which is reflected as cognitive deterioration and increased release of pro-inflammatory factors in the hippocampus (TNF-α, IL-1β, and IL-6). Subsequent mechanistic research has validated that miR-138–5p may target and influence the activity of the lipocalin 2 (LCN2) gene, and the use of isoflurane anesthesia leads to a significant increase in LCN2 expression, which triggers neuroinflammatory hippocampal responses (Lou et al., 2025). This implies that downstream target genes and inflammatory pathways can be controlled through anesthetic agents by modulating the expression of certain miRNAs, which ultimately affects cognitive functions.

**FIGURE 3:**
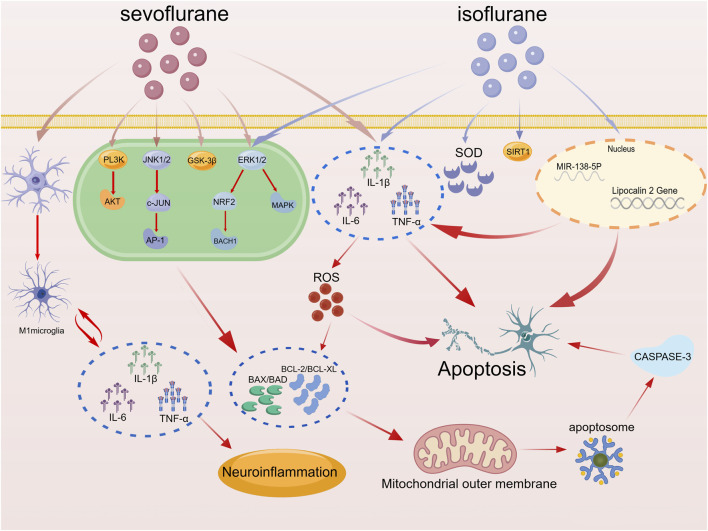
Overview of the pathological mechanisms of anesthesia-induced cognitive dysfunction. Akt, Protein Kinase B; AP-1, activating protein-1; BACH1, BTB domain and CNC homolog 1; Bad, Bcl2-Associated Agonist of Cell Death Protein; Bax, Bcl2-Associated X Protein; c-JUN, Jun Proto-Oncogene; BCL-2, B-cell lymphoma 2; BCL-XL, B-cell lymphoma-extra large; CASPASE-3, Cysteinyl Aspartate Specific Proteinase-3; ERK1/2, Extracellular-Signal-Regulated Kinase 1/2; GSK-3β, Glycogen Synthasekinase-3β; IL-1β, Interleukin-1beta; IL-6, Interleukin-6; JNK, c-Jun N-terminal Kinase; LCN2, Lipocalin 2; MAPK, Mitogen-Activated Protein Kinases; NRF2, Nuclear Factor Erythroid 2-Related Factor 2; PI3K, Phosphoinositide 3-Kinase; ROS, Reactive Oxygen Species; SIRT1, A Homologous Family of Regulatory Enzymes Containing 3; SOD, Superoxide Dismutase; TNF-α, Tumor Necrosis Factor Alpha.

Current animal experiments predominantly use a single exposure (2–6 h) or repeated exposure (e.g., 2 h daily from P6-P8) of isoflurane (0.75%–3%) or sevoflurane (2%–4.1%). Under these anesthetic conditions, the induced cognitive dysfunction involves various pathological mechanisms such as neuroinflammation, oxidative stress and neuronal apoptosis, microglial polarisation, and miRNA/epigenetic regulation. These mechanisms do not exist in isolation but form a complex interactive network: neuroinflammation can directly induce oxidative stress, which in turn activates the mitochondrial apoptosis pathway, leading to neuronal damage. In this network, microglial polarization plays an ‘amplifier’ role—M1-type polarization exacerbates inflammation and oxidative damage, whereas M2-type polarization is supposed to exert a protective effect, but its function is often inhibited by anesthetic drugs. Furthermore, miRNAs and epigenetic modifications are located at the upstream regulatory level, capable of simultaneously affecting the expression of inflammatory factors and regulating the translation of apoptosis-related proteins, thereby finely modulating the downstream pathways. Figure 3 intuitively demonstrates the hierarchical relationships and interactive effects among these mechanisms, providing an integrated framework for understanding the complex pathological network of anesthesia-induced cognitive dysfunction.

## 4 Research progress about mechanisms of natural compounds in treating anesthesia-induced cognitive dysfunction

### 4.1 Research on natural compounds related to anti-inflammatory mechanisms

In the research on the treatment of cognitive dysfunction caused by anesthesia with natural compounds, the anti-inflammatory mechanism is one of the important pathways. Various natural compounds have effectively alleviated cognitive dysfunction induced by anesthetics by inhibiting neuroinflammatory responses and regulating related signaling pathways.

Tanshinone IIA (Tan IIA), as the main lipid-soluble metabolite of *Salvia miltiorrhiza* Bunge [Lamiaceae] (i.e., *Danshen*, a traditional Chinese medicine), possesses significant anti-inflammatory, anti-apoptotic, and antioxidant properties. Research shows that continuous intraperitoneal injection of Tan IIA in neonatal mice can significantly alleviate sevoflurane-induced cognitive dysfunction. The mechanism of action underpinning the therapeutic effect is focused on neuroinflammatory suppression, which is manifested by significant inhibition of excessive expression of pro-inflammatory mediators (IL-1β and IL-6) in the hippocampal regions and at the same time decreasing neuronal apoptosis and preserving the structural integrity of the postsynaptic density in the hippocampus, ultimately improving contextual fear memory deficits in mice (Xia et al., 2017).

Gynosaponin (GpS) has been shown to exhibit cognitive protective effects and anti-inflammatory effects by regulating the PI3K/Akt/mammalian target of rapamycin (mTOR) signaling cascade. *In vitro* and *in vivo* research shows that GpS can significantly inhibit the production of anesthetic-induced pro-inflammatory cytokines (TNF-α and IL-6) and reduce neuroinflammatory and oxidative stress-induced injuries. The molecular mechanism involves the activation of phosphorylation of the PI3K/Akt/mTOR pathway, thereby inhibiting neuronal apoptosis. Furthermore, the use of pathway inhibitors can reverse the protective effects of GpS, confirming the critical role of the PI3K/Akt/mTOR signaling pathway in the anti-inflammatory mechanism of GpS (Lin et al., 2024).

Morroniside (Mor), derived from *Cornus officinalis* Siebold & Zucc [Cornaceae], suppresses anesthesia-induced neuroinflammation by specifically targeting and inhibiting the TLR4/NF-κβ signaling pathway. Studies indicate that Mor can dose-dependently alleviate learning and spatial memory deficits induced by sevoflurane in aged mice. The therapeutic effect is mediated by a decrease in the expression of inflammatory mediators (TNF-α, IL-1β, IL-6) in hippocampal regions and the promotion of microglial phenotypic switching (inhibition of M1 polarization markers clusters of differentiation 86 (CD86) and inducible nitric oxide synthase (iNOS), and production of M2 polarization markers clusters of differentiation 206 (CD206) and arginase-1 (Arg-1) to induce anti-inflammatory effects. Molecular docking experiments verify that Mor has high affinity for TLR4, which can prevent the activation of the TLR4/NF-κβ pathway, and TLR4 agonists can partially negate its protective effects (Chen et al., 2025).

Moreover, Chikusetsu saponin IVa (ChIV) attenuates neuroinflammation by inhibiting the activation of the NOD-like receptor 3 (NLRP3) inflammasome. The experimental results suggest that ChIV can suppress sevoflurane-induced expression of pro-inflammatory factors (IL-6, IL-1β, TNF-α) and cellular apoptosis in the structures of the hippocampus. The underlying mechanism involves the inhibition of the expression of NLRP3 inflammasome-related proteins (NLRP3, apoptosis-associated speckle-like protein (ASC), and caspase-1) and the release of downstream pro-inflammatory factors IL-1β and interleukin-18 (IL-18). *In vitro* research also confirms the ability of the compound to reduce neuronal apoptosis and inflammatory responses (Shao et al., 2020).

The aforementioned natural compounds inhibit the release of pro-inflammatory factors and regulate the immune cell phenotype through different signaling pathways (such as TLR4/NF-κB, PI3K/Akt/mTOR, NLRP3 inflammasome, etc.), thereby reducing anesthetic-induced neuroinflammation. From the perspective of interactions between these mechanisms, TLR4/NF-κβ occupies an upstream core position, and its activation can directly induce the activation of the NLRP3 inflammasome. Meanwhile, the PI3K/Akt pathway can negatively regulate NF-κβ. Therefore, TLR4/NF-κβ should be considered the core anti-inflammatory mechanism, whereas PI3K/Akt/mTOR and NLRP3/caspase-1 belong to auxiliary or downstream execution pathways. This hierarchical relationship provides a theoretical basis for understanding the primary and secondary status of different natural compounds in anti-inflammatory mechanisms and suggests the strategic value of prioritizing the targeting of upstream core pathways in combination therapy.

### 4.2 Study of natural compounds related to antioxidant mechanisms

Natural antioxidants play an important role in addressing oxidative stress and related cognitive dysfunction caused by anesthesia, among which flavonoids are a research hotspot. Baicalein, as a key natural flavonoid compound, demonstrates significant antioxidant and neuroprotective effects, capable of reducing neuronal apoptosis induced by sevoflurane exposure in rats and improving cognitive and memory functions. Its mechanism involves regulating the JNK/ERK signaling pathway, inhibiting pro-apoptotic proteins Bax and Bad, and enhancing the expression of anti-apoptotic proteins Bcl-2, Bcl-xL, and apoptosis-inhibiting factors (X-linked inhibitor of apoptosis protein (xIAP), cIAP-1, cIAP-2, and survivin), while also activating the PI3K/Akt/GSK-3β signaling pathway (Wang and Zhou, 2018), increasing the expression of Akt and its phosphorylated form, and promoting the upregulation of cell survival-related proteins cyclin D1, p-NF-κβ, p65, and p-IκBα(Wang and Zhou, 2018).

Rutin, a flavonoid compound, significantly inhibits isoflurane-induced apoptosis of neuronal cells in the hippocampal region of neonatal rats by regulating the MAPK signaling pathway (including JNK, ERK, and p38). Experimental evidence shows that rutin reduces the number of apoptotic cells (TUNEL-positive cells), the expression levels of activated caspase-3, and the expression of pro-apoptotic proteins Bax and Bad in a dose-dependent manner, while upregulating the expression of anti-apoptotic proteins Bcl-2, Bcl-xL, and phosphorylated Bad. Furthermore, it improves the learning and memory abilities of rats exposed to isoflurane, shortening the escape latency in the Morris water maze test (Li et al., 2017a).

Luteolin can mitigate anesthesia-induced cognitive dysfunction by inhibiting oxidative stress and inflammatory responses. Its pretreatment alleviates sevoflurane-induced apoptosis of hippocampal neurons, as evidenced by a reduction in TUNEL-positive cells, decreased cleavage levels of caspase-3 and poly-adenosine diphosphate-ribose polymerase (PARP), and inhibition of their activity. Furthermore, quercetin significantly reduces the levels of ROS and the lipid peroxidation product malondialdehyde in hippocampal tissue, enhances the activity of SOD, and improves spatial learning and memory impairments in sevoflurane-anesthetized animals by downregulating the expression of proteins related to the NF-κβ/NLRP3 inflammasome pathway (NF-κβ, NLRP3, caspase-1, and ASC) and inhibiting NF-κβ activity, thereby reducing the expression of inflammatory factors IL-18, IL-1β, and TNF-α(Wang et al., 2019).

Apart from flavonoids, the main active metabolite of *Panax ginseng* C. A. Mey [Araliaceae], ginsenoside Rg1, also improves anesthesia-induced cognitive dysfunction through its antioxidant effects. It significantly reduces the level of malondialdehyde in the hippocampal tissue of elderly rats induced by isoflurane, inhibits oxidative stress, and simultaneously restores glutathione levels. The neuroprotective effect of ginsenoside Rg1 is also associated with the activation of the PI3K/AKT/GSK-3β signaling pathway, which exerts anti-inflammatory and anti-apoptotic effects by increasing the ratio of p-AKT/AKT and the expression of GSK-3β protein, while downregulating the expression of p21WAF1/CIP1 and p53 proteins (Zhang et al., 2016).

The aforementioned multiple compounds primarily act through three main antioxidant pathways: PI3K/Akt/GSK-3β, JNK/ERK MAPK, and NF-κβ/NLRP3. From the perspective of mechanistic interactions, PI3K/Akt is an upstream core node that regulates cell survival, and its activation can inhibit JNK and NF-κβ signaling; therefore, the PI3K/Akt pathway should be considered the core antioxidant/anti-apoptotic mechanism. JNK/ERK and NF-κβ/NLRP3, meanwhile, are auxiliary pathways mediating mitochondrial apoptosis and oxidative damage related to inflammation, respectively. Among the different compounds, baicalein simultaneously activates PI3K/Akt and inhibits JNK/ERK, providing dual regulation of the core and auxiliary pathways, thus possessing the broadest antioxidant spectrum. In contrast, rutin mainly inhibits JNK/p38, resulting in a relatively narrower scope of action. Therefore, among the compounds currently under discussion, Baicalein, by acting on both the core and auxiliary pathways, possesses the broadest spectrum of antioxidant protection, while compounds such as rutin may be more suitable as metabolites of adjuvant or combination therapies. This difference suggests that baicalein may hold a greater advantage in antioxidant strategies.

### 4.3 Inhibition of neuronal apoptosis and cell protection mechanisms

Natural compounds can exert protective effects on cells and improve cognitive dysfunction by regulating the expression of apoptosis-related proteins and activating cell survival signaling pathways, thereby inhibiting anesthesia-induced apoptosis of nerve cells. For instance, paeonol can significantly suppress isoflurane-induced neurotoxicity in the hippocampus of neonatal rats, reducing neuronal apoptosis. This is specifically manifested by the downregulation of pro-apoptotic proteins cleaved caspase-3, Bad, and Bax, which are upregulated after isoflurane exposure, while enhancing the expression of anti-apoptotic proteins Bcl-2, Bcl-xL, xIAP, c-IAP-1, c-IAP-2, and survivin, and promoting neuronal survival through the modulation of the JNK/ERK/p38MAPK signaling pathway (Jin et al., 2020). Chikusetsu saponin IVa (chIV) pretreatment can alleviate isoflurane-induced neurotoxicity and spatial memory impairment in neonatal rats, and its mechanism is associated with the upregulation of SIRT1/ERK1/2 signaling pathway expression. This effect can suppress neuronal apoptosis by increasing the expression of the anti-apoptotic protein Bcl-2 and reducing the expression of the pro-apoptotic protein Bax. Moreover, the SIRT1 inhibitor sirtinol can counteract the chIV-induced upregulation of SIRT1 and p-ERK1/2 expression and the protective effect on cells, suggesting that the neuroprotective effect of chIV may be mediated by the SIRT1/ERK1/2 pathway (Fang et al., 2017). Hispidulin, a natural flavonoid compound, can induce autophagy by activating the AMP-activated protein kinases (AMPK) signaling pathway, thereby inhibiting neuronal apoptosis caused by the inhalational anesthetic sevoflurane. For instance, in the human neuroglioma cell line H4, the increase in pro-apoptotic proteins cleaved caspase-3 and Bax, and the decrease in the anti-apoptotic protein Bcl-2 induced by sevoflurane treatment can be reversed by pretreatment with hispidulin. Additionally, silencing the AMPKα gene using siRNA significantly blocks the autophagy induced by hispidulin and its protective effect against neuronal apoptosis. Animal experiments have also shown that hispidulin pretreatment can alleviate cognitive dysfunction and neuronal apoptosis in the hippocampus induced by sevoflurane in aged rats (Huang et al., 2018). Furthermore, eleutheroside E may participate in the cellular protective process by increasing the expression of α7-nicotinic acetylcholine receptors (α7-nAChR)/NR2B and regulating the cholinergic system to reverse the behavioral abnormalities induced by isoflurane, thereby improving cognitive dysfunction induced by anesthesia (Lu and Xiao-Qing, 2019).

Overall, the aforementioned compounds primarily involve four upstream pathways: JNK/ERK/p38 MAPK, SIRT1/ERK1/2, AMPK/mTOR autophagy, and α7-nAChR/NMDAR. From the perspective of mechanism hierarchy, the mitochondrial apoptosis pathway (Bcl-2/Bax/caspase-3) is the final common execution point for all upstream signals and should be considered the core mechanism; the others are regulatory auxiliary pathways. Among the different compounds, Paeonol exhibits the most comprehensive regulation of apoptotic proteins (downregulation of Bax/Bad, upregulation of Bcl-2/Bcl-xL), directly acting upon the core apoptotic execution site and demonstrating the strongest effect. The efficacy of other compounds is lesser, while eleutheroside E exerts its protection through the cholinergic pathway, making its action the most indirect. This hierarchical relationship of mechanisms suggests that in anti-apoptotic intervention strategies, preferentially targeting the core mitochondrial apoptosis pathway may be more efficient than merely modulating upstream auxiliary pathways. Moreover, due to its direct regulatory effect on the execution site, Paeonol shows significant anti-apoptotic potential.

### 4.4 Microglial polarization and neuroimmune regulation mechanisms

Cognitive dysfunction caused by anesthesia is often accompanied by neuroinflammatory responses. In the rat cognitive dysfunction model induced by isoflurane, the levels of pro-inflammatory cytokines in the hippocampus were significantly elevated, suggesting that microglia may shift towards a pro-inflammatory M1 polarization state. Curcumin, as a natural compound, can significantly improve isoflurane-induced cognitive dysfunction, with its mechanism closely related to the inhibition of neuroinflammation, manifested as a reduction in the release of pro-inflammatory cytokines. This process involves the regulation of miR-181a-5p: miR-181a-5p is upregulated in the isoflurane-induced model, while curcumin can reduce its expression level. Subsequent investigations indicate that overexpression of miR-181a-5p facilitates isoflurane-induced cognitive dysfunction and the release of pro-inflammatory cytokines. Suppressing this microRNA can reduce cognitive dysfunction and neuroinflammation, which suggests that curcumin may suppress the polarization of M1 microglia by regulating the expression of miR-181a-5p, thus preventing neuroinflammation and enhancing cognitive function (Liu et al., 2021).

Picroside II, similarly, has been demonstrated to restore isoflurane-induced cognitive dysfunction in rats, reducing the escape latency and extending the duration of stay in the target quadrant, and its effect is mediated by suppression of neuroinflammation. After isoflurane treatment, the expression of pro-inflammatory cytokines increases in rat hippocampal tissue and neurons, while the level of miR-195 significantly upregulates; however, Picroside II can reduce the expression of pro-inflammatory cytokines and miR-195. Further experiments indicate that the overexpression of miR-195 enhances the release of pro-inflammatory cytokines, promotes neuronal apoptosis, and reduces cell viability, thereby reversing the protective effects of Picroside II. This suggests that Picroside II may protect cognitive function by downregulating miR-195 and inhibiting excessive activation of microglia and inflammation related to M1-type cell polarization (Li et al., 2022a).

In addition to regulating microRNA-mediated inflammatory pathways, natural compounds can also influence cognitive function by modulating autophagy-related immune pathways. Hispidulin, as a natural flavonoid, exerts neuroprotective effects in the isoflurane-induced cognitive dysfunction model by activating the AMPK signaling pathway to induce autophagy. In H4 cells, hispidulin regulates the expression of autophagy-related proteins LC3 II and p62, significantly enhancing sevoflurane-induced autophagy, and the use of autophagy inhibitors can weaken its cytoprotective effects. AMPKα gene silencing experiments further confirm that the AMPK signaling pathway is crucial for hispidulin-induced autophagy and anti-apoptotic functions. In an aging rat model of sevoflurane-induced cognitive dysfunction, hispidulin promotes autophagy by activating AMPK and inhibiting mTOR, which not only reduces hippocampal neuronal apoptosis but also significantly improves cognitive function, reflecting its potential mechanism of involvement in neuroimmune regulation through the modulation of the AMPK/mTOR autophagy pathway, thereby affecting cognitive function (Huang et al., 2018).

The natural compounds mentioned above mainly work by regulating miR-181a-5p and miR-195 to inhibit M1 type microglial polarization and promote M2 type polarization, thereby reducing the release of inflammatory factors such as TNF-α, IL-1β, and IL-6. Hispidulin, on the other hand, induces autophagy by activating the AMPK/mTOR pathway, alleviating neuroinflammation and cognitive dysfunction. From the perspective of interactions between mechanisms, the M1/M2 polarization balance of microglia, miRNA regulation (miR-181a-5p, miR-195), and the autophagy pathway (AMPK/mTOR) are involved in neuroimmune regulation at different levels, and they may collectively influence the outcome of neuroinflammation in a relatively independent, parallel, and synergistic manner. Among the different compounds, only Hispidulin exerts a protective effect indirectly through autophagy, with a weaker direct inhibitory effect on inflammatory factors. However, these differences do not imply that a certain mechanism or compound is central, but rather reflect the complementarity of different intervention strategies in terms of their action points and effect spectra. Therefore, there is no clear primary or secondary distinction between the above mechanisms and compounds. They each play a role with different focuses in microglial polarization and neuroimmune regulation, and their combined application may have a broader anti-inflammatory effect than a single intervention.

### 4.5 miRNA and epigenetic regulatory mechanisms

Some natural compounds can improve anesthesia-induced cognitive dysfunction by regulating microRNA expression and epigenetic modifications. In a rat model of cognitive dysfunction induced by isoflurane anesthesia, quercetin treatment can enhance cognitive function and suppress the levels of inflammatory factors in the hippocampus, with its mechanism related to the regulation of miRNA expression: isoflurane resulted in a significant downregulation of miR-138–5p expression in the hippocampus of rats, while quercetin treatment significantly elevated miR-138–5p levels. Further experiments validated the direct binding of miR-138–5p to its target gene LCN2 through dual-luciferase reporter assays. LCN2 was markedly upregulated under isoflurane induction, while quercetin inhibited LCN2 expression, and this inhibitory effect could be reversed by the knockdown of miR-138–5p. This indicates that quercetin may suppress isoflurane-induced hippocampal neuroinflammation by regulating the miR-138–5p/LCN2 pathway, thereby improving cognitive dysfunction (Lou et al., 2025).

In terms of epigenetic modifications, resveratrol exhibits a protective effect against sevoflurane-induced cognitive dysfunction, with mechanisms involving the regulation of the histone deacetylase SIRT1: repeated exposure to 3% sevoflurane leads to a significant downregulation of SIRT1 expression in the hippocampus of developing mice, accompanied by a notable increase in NF-κβ acetylation levels and nuclear NF-κβ levels, along with activation of microglia and elevated expression of pro-inflammatory cytokines IL-6 and TNF-α, resulting in impaired cognitive function. Resveratrol, as a SIRT1 agonist, restores the expression level of SIRT1, inhibits the acetylation and nuclear translocation of NF-κβ, regulates the M1/M2 phenotypic ratio of microglia (increasing CD206 mRNA levels and decreasing CD86 and suppressor of cytokine signaling 3 (SOCS3) mRNA levels), and suppresses neuroinflammation. Its role in ameliorating sevoflurane-induced cognitive deficits is closely related to its epigenetic regulation of SIRT1-dependent mechanisms (Tang et al., 2021).

The two aforementioned compounds belong to different levels of gene expression regulation. At present, there is no direct evidence that SIRT1 regulates miR-138–5p, and the two pathways may participate in the pathological process of anesthesia-induced cognitive dysfunction relatively independently and in parallel. The difference in the breadth of their spectrum of action only reflects the characteristics of their respective targets and does not constitute a primary-secondary distinction. In the future, the possibility of their combined application can be explored, which also provides a noteworthy direction for subsequent research.

**FIGURE 4:**
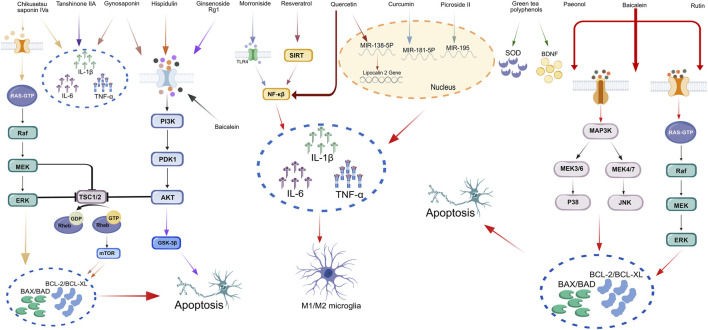
Overview of the primary mechanisms of various natural compounds. Akt, Protein Kinase B; Bad, Bcl2-Associated Agonist of Cell Death Protein; Bax, Bcl2-Associated X Protein; c-JUN, Jun Proto-Oncogene; BCL-2, B-cell lymphoma 2; BCL-XL, B-cell lymphoma-extra large; ERK1/2, Extracellular-Signal-Regulated Kinase 1/2; GSK-3β, Glycogen Synthasekinase-3β; IL-1β, Interleukin-1beta; IL-6, Interleukin-6; JNK, c-Jun N-terminal Kinase; LCN2, Lipocalin 2; MAPK, Mitogen-Activated Protein Kinases; mTOR, Mammalian Target of Rapamycin; NF-кβ, MEK, Mitogen-Activated Protein Kinase; Nuclear Factor Kappa β; PDK1, Phosphoinositide-dependent kinase-1 PI3K, Phosphoinositide 3-Kinase; P38, P38 mitogen-activated protein kinase; Raf, Rapidly Accelerated Fibrosarcoma Kinase; RAS-GTP, Ras-Specific GTPase-Activating Proteins; Rheb, Ras Homolog Enriched in Brain; SIRT, A Homologous Family of Regulatory Enzymes Containing 3; SOD, Superoxide Dismutase; TLR4, Toll-Like Receptor 4; TNF-α, Tumor Necrosis Factor Alpha; TSC1/2, Tuberous Sclerosis Complex 1/2.

The intervention of these natural compounds involves multiple signaling pathways, but different signaling pathways do not operate in isolation; instead, they form complex regulatory networks. The characteristics of each compound’s action also show differences: baicalein and paeonol can act simultaneously on core apoptotic nodes and their upstream pathways, demonstrating a more comprehensive protective effect. Resveratrol regulates both inflammation and the polarization of microglia by activating SIRT1; compounds such as curcumin and quercetin primarily exert auxiliary protective effects by regulating miRNA (such as miR-181a-5p, miR-138–5p) or autophagy pathways; hispidulin and gynosaponin induce autophagy through the AMPK/mTOR pathway. Figure 4 visually integrates the corresponding relationship between the aforementioned compounds and signaling pathways: from the upstream TLR4, PI3K, MAPK, to the midstream NF-κβ, GSK-3β, mTOR, and further to the downstream Bcl-2 family and various inflammatory factors, natural compounds block anesthetic-induced neurotoxicity in a multi-layered and multi-target manner, providing a theoretical basis for combination therapy and personalized treatment strategies.

## 5 Basic research and clinical practice

Despite the fact that animal models are the fundamental basis of investigating the cognitive processes influenced by anesthetics, there are substantial challenges in their research results in translating the findings to the more complex and variable clinical situations. Animal models that are highly standardized have difficulties in completely replicating the enormous heterogeneity of human patient populations in terms of age, genetic background, type of surgical procedure, and comorbidity. Consequently, the mechanistic studies that rely on the animal experiments are rather comprehensive, but the processed data that can directly inform clinical practice are rather scarce. Moreover, we established that the molecular specificity of the actions of anesthetics on cognitive dysfunction, the specific dose-response relationship, and the multifaceted interactions with personal risk factors have never been properly confirmed in animal and clinical studies, which highly undermines the clinical translation success rate of new drugs and new therapeutic solutions developed on the basis of the results of experiments in animals (Luo et al., 2025).

Furthermore, natural compounds such as resveratrol, quercetin, and curcumin have shown potential in neuroprotection (Ogunro, 2025; Rastogi et al., 2025; Mattioli et al., 2026), but existing research has significant limitations. Multiple studies are constrained by small sample sizes, leading to results that are difficult to generalize. For example, clinical trials of resveratrol often involve small sample sizes, a wide range of doses, and diverse populations, making it difficult to determine the safe and effective dose range for specific populations (Novelle et al., 2015). In clinical trials of curcumin and resveratrol, the number of subjects was generally small, and the follow-up periods were too short to detect potential changes in cognitive function. Furthermore, the dosage of curcumin varied greatly, ranging from 80 mg/day to 4 g/day, with low doses improving behavioral symptoms whereas high doses showed no positive effects (Mazzanti and Di Giacomo, 2016). Many preclinical studies use unrealistic conditions, such as excessively high concentrations, short-term exposure, and non-physiological metabolites, which limit the extrapolation to humans; at the same time, the paradox of resveratrol’s ‘low bioavailability but high bioactivity’ remains unresolved, and the final target sites responsible for the effects have not yet been identified (Tomé-Carneiro et al., 2013). The current research included studies with low methodological quality, lacked sample size calculations, and had a high risk of bias, and these defects inevitably affect the correctness and generalizability of the research conclusions (Chen et al., 2019). In vivo studies, the results are often controversial. For example, some studies show that quercetin has neuroprotective effects, while other studies have not observed this, requiring drug delivery systems or chronic treatment to manifest effects, which is related to its low bioavailability in the blood and brain, and high concentrations even exhibit cytotoxicity and apoptotic effects (Dajas et al., 2015).

Preclinical research of natural compounds in the field of neuroprotection has made significant progress (Suswidiantoro et al., 2026), with a variety of compounds demonstrating application potential through improved bioavailability and the conduct of targeted clinical trials. Curcumin, as one of the most extensively studied natural compounds, currently has its clinical translation primarily focused on overcoming the challenge of low bioavailability. Various nanomaterials have significantly enhanced oral bioavailability; for instance, micellar curcumin in healthy subjects increased the area under the curve by 14 to 277-fold, whereas incorporating curcumin into micelles via the solvent evaporation method enhanced water solubility by 10^3^ to 10^4^-fold and increased the area under the curve by seven to 13-fold (Yallapu et al., 2015; Liu et al., 2016; Ma et al., 2019). In terms of cognitive function improvement, Longvida® Optimized Curcumin (400 mg/day) significantly enhances sustained attention and working memory in healthy older adults, with an acute administration showing an effect within 1 h (Mazzanti and Di Giacomo, 2016). In inflammatory diseases, a daily dose of 80 mg of curcumin nano-micelle can significantly improve C-reactive protein and TNF levels (Peng et al., 2021).

The clinical translation of resveratrol faces challenges in dose standardization and safety. The dose range reported in current clinical trials is wide (5 mg–5 g), and the sample sizes are generally small. There is an urgent need to determine the minimum effective concentration to balance health benefits with side effects (Novelle et al., 2015). In the field of cognitive function, class II evidence provided by the study of Turner et al. showed that resveratrol (500 mg/day to 2 g/day) is well-tolerated, safe, and able to decrease A*β*_40_ levels in cerebrospinal fluid and plasma but has no significant effects on cognitive score (Turner et al., 2015). Conversely, Witte et al. found in overweight, healthy older adults that 200 mg/day of resveratrol combined with quercetin for 26 weeks enhanced memory retention and hippocampal functional connectivity (Witte et al., 2014).

Strategies for the transformation of natural compounds deserve serious attention. For example, the development of novel drug delivery systems (such as liposomal delivery systems for curcumin) can prolong *in vivo* retention time and increase encapsulation efficiency (Chopra and Dhingra, 2021), the clinical application of curcumin in cardiovascular diseases, osteoarthritis, and Crohn’s disease has confirmed its safety and efficacy, providing a reference for translation in the cognitive field (Fu et al., 2021). At the same time, the patented technology for resveratrol drug delivery formulations is driving the optimisation of its bioavailability (Harwansh et al., 2023). These advancements collectively accelerate the translation of natural compounds into cognitive dysfunction treatments.

## 6 Discussion

The association between anesthetic agents and cognitive dysfunction has become an important focus of perioperative medical research. Although huge strides have been achieved in this area by the use of animal models in unraveling the possible mechanisms, there is still a substantial gap between basic research and clinical practice. The limitations of research that are presently experienced are mainly manifested in three ways: the challenge in extrapolating animal models into clinical practice, the lack of sufficient depth in the exploration of mechanisms, and the barriers to clinical implementation of natural compounds despite their promise.

### 6.1 Assessment of potential Pan-Assay Interference Compounds (PAINS) and non-specific bioactivity

When evaluating the pharmacological effects of natural compounds on inhalation anesthesia-induced cognitive dysfunction, it is essential to address a well-recognized issue in drug discovery: Pan-Assay Interference Compounds (PAINS). PAINS were originally derived from compounds that frequently appeared as false-positive hits in high-throughput screenings, based on which Baell and Holloway defined 480 PAINS-defining substructures (Baell and Holloway, 2010). However, the mechanisms underlying PAINS interference have long been incompletely understood—whether their activity arises from chemical reactivity or non-specific binding has remained a subject of debate.

A structural perspective on PAINS binding modes. Bolz systematically analyzed the binding modes of 871 PAINS-containing ligands and 517 protein targets in the Protein Data Bank, classifying 34 PAINS classes by calculating the Tanimoto interaction similarity (TIS) of their non-covalent interaction fingerprints (Bolz et al., 2021). Their study yielded two key findings. First, different PAINS classes exhibit markedly different levels of binding mode conservation—for example, rhodanine derivatives and azo compounds display highly conserved binding modes, whereas catechols and Mannich bases show highly variable binding modes. Second, PAINS classes with variable binding modes can still bind multiple targets with nanomolar affinity. These findings suggest that the “apparent activity” of PAINS may arise in part from structure-encoded non-covalent binding promiscuity, rather than from chemical reactivity or assay artifacts alone.

Membrane PAINS: non-specific effects through bilayer perturbation. In addition to direct target engagement, certain PAINS can alter membrane protein function by non-specifically perturbing the lipid bilayer; such compounds are referred to as “membrane PAINS”. Molecular dynamics simulations enable the quantitative assessment of a compound’s membrane perturbation potential by calculating its effect on lipid bilayer deformation propensity—an approach that has been successfully used to identify membrane PAINS(Magalhães et al., 2021). Study revealed that curcumin markedly reduces the deformation energy barrier (at approximately 1.8 nm), exhibiting typical membrane PAINS behavior, whereas resveratrol shows a relatively mild membrane-perturbing effect (Ingólfsson et al., 2014; Magalhães et al., 2021).

PAINS risk assessment of the compounds covered in this review. According to the classification framework of Bolz and known PAINS alert substructures, several compounds in Table 1 of this review carry potential PAINS risk: High-risk catechols–quercetin, luteolin, and rutin contain ortho-dihydroxyphenol moieties corresponding to the catechol_A alert substructure, which exhibits a highly variable binding mode (TIS = 0.06) and readily engages in non-specific interactions with multiple protein targets. High-risk quinones–tanshinone IIA contains a quinone structure corresponding to the quinone_A class (TIS = 0.09). High-risk α,β-unsaturated ketones–curcumin contains a Michael acceptor and has been confirmed as a membrane PAINS, while resveratrol contains a stilbene structure. Moderate-risk polyphenols–baicalein and green tea polyphenols, although not exactly matching a specific PAINS alert substructure, still carry potential redox activity risks due to their polyphenolic structures. By contrast, compounds such as hispidulin, morroniside, chikusetsu saponin IVa, ginsenoside Rg1, and paeonol are associated with lower PAINS risk.

Evidence interpretation strategy of this review. Based on the PAINS risk assessment above, this review adopts a three-tiered evidence interpretation strategy (Table 3). First, *in vivo* and clinical studies are prioritized as core evidence because they are least susceptible to PAINS artifacts. Second, cellular-level studies are used only as supportive evidence. Third, purely biochemical *in vitro* studies—especially those involving PAINS-suspect metabolites—are treated solely as hypothesis-generating observations and are not used as core evidence. Through this evidence hierarchy, the review aims to provide a more critically framed and reliable evaluation of the existing literature.

**TABLE 3:** Evidence hierarchy and data interpretation principles adopted in this review.

Level of evidence	Definition and scope	Handling strategy in this review	Strength of conclusion	PAINS risk consideration
High (*in vivo*/clinical)	Animal model efficacy studies; trials/observations; Known *in vivo* metabolism/PK data	Highlighted as primary evidence for conclusions	High	Minimal PAINS concern; prioritized
Medium (cellular/tissue)	Functional assays in intact cell lines Isolated tissue/brain slice studies	Used cautiously as supportive evidence only	Moderate	Moderate concern; interpreted with caution
Low (*in vitro* biochemical)	Purified enzyme inhibition assays Molecular interaction analyses Fluorescence/colorimetric direct assays Single-concentration screens without cytotoxicity controls	Used critically and only as hypothesis-generating	Low	Highest PAINS risk; must be interpreted with appropriate controls

### 6.2 Anesthesia and surgery on cognitive dysfunction

The pathogenesis of cognitive dysfunction involves complex interactions among multiple factors, such as surgical trauma, systemic inflammatory response, exposure to anesthetic drugs, and patient age, rather than being caused by a single factor. Surgical trauma can lead to the release of damage-associated Molecular Patterns, activating the NF-κβ signaling pathway in immune cells, which in turn releases various cytokines and chemokines; simultaneously, the upregulation of cyclooxygenase two and matrix metalloproteinases increases blood-brain barrier permeability, allowing peripheral pro-inflammatory cytokines and macrophages to enter the central nervous system, activating microglia and amplifying neuroinflammation (Rump and Adamzik, 2022). However, anesthetic drugs can also activate neuroinflammatory pathways, thereby significantly increasing the release of pro-inflammatory cytokines. These cytokines, after penetrating the blood-brain barrier, activate neutrophils in the central nervous system. As key effector cells of the innate immune system, neutrophils play a crucial role in cognitive dysfunction. They directly degrade tight junction proteins of the blood-brain barrier by releasing myeloperoxidase and elastase, induce endothelial cell apoptosis, and promote the infiltration of matrix metalloproteinase-9 into brain tissue, thereby exacerbating blood-brain barrier damage and neuroinflammation (Cheng et al., 2025). This further amplifies neuroinflammation and impairs neurological function, ultimately leading to cognitive dysfunction.

In terms of anesthetic drugs, research has shown that anesthetics can mediate cognitive dysfunction by inducing the release of inflammatory mediators, oxidative stress, cellular apoptosis, and epigenetic mechanisms; anesthetics regulate the expression of inflammation-related and neurodevelopment-related genes through the modulation of DNA methylation, histone modification, and non-coding RNA regulation. However, animal experimental evidence suggests that surgery itself, rather than simple anesthetic exposure, is the primary driving factor of cognitive dysfunction. For example, studies show that the risk of cognitive dysfunction is higher with surgery than with anesthetic exposure alone; in the absence of general anesthesia, abdominal surgery alone can induce cognitive dysfunction in 18-month-old wild-type mice. In young animals, simple anesthetic exposure often inconsistently affects cognitive function, while results in old animals are conflicting; repeated anesthetic exposure may increase risk, especially in Alzheimer’s disease transgenic models (Belrose and Noppens, 2019).

Clinical research has further revealed the complexity of the effects of anesthesia and surgery. Patient characteristics, especially age, are important risk factors for cognitive dysfunction. In elderly patients over 60 years of age, the incidence of cognitive dysfunction at 3 months after major non-cardiac surgery can be as high as 39%, which is significantly higher than in patients under 60 years of age. Furthermore, individual patient characteristics, type of surgery, anesthetic method, and pain level are all considered to be closely related to the occurrence of cognitive dysfunction; The incidence of cognitive dysfunction is much higher in major abdominal and hip fracture surgery than in superficial minor operations. Surgery itself can increase the platelet/lymphocyte ratio, while the increase in the neutrophil/lymphocyte ratio is only observed in patients with cognitive dysfunction (Zhao et al., 2023). In gastric cancer surgery, some studies have mainly attributed cognitive dysfunction to abnormal central nervous system stimulation by residual anesthetic agents, and the etomidate combined with the propofol regimen can effectively reduce the expression of inflammatory factors and has a lesser impact on cognitive function (Xiuwen, 2023).

The current consensus holds that while cognitive dysfunction may be caused by multiple factors, surgery is the main cause of cognitive dysfunction. Clinical studies show that there is no difference in cognitive performance within 3 months post-operation between general anesthesia and regional anesthesia. Therefore, the choice of anesthesia may not affect the incidence of cognitive dysfunction. However, controversies still remain, such as different types of surgery, the choice of anesthetic, and the potential risk of repeated anesthetic exposure (Luo et al., 2025).

### 6.3 Exploration of the mechanism

There are obvious shortcomings in the depth of the current mechanism research itself. There is limited information on how anesthetic agents specifically act on various molecular sites and how their action is also related to the individual risk factors in patients. The particular signaling pathway networks of cognitive dysfunction after anesthesia have not been identified in a clear and exhaustive manner, and thus it is difficult to create effective interventions to focus on the major nodes (Aktary et al., 2025). Indicatively, resveratrol has neuroprotective actions mediated by regulating the SIRT1/NF-κβ axis, although the possible activities of other sirtuins family members (such as SIRT2) remain unclear. Insufficient mechanism research may lead to difficulties in precise targeting during clinical applications, increasing uncertainty in efficacy (Tang et al., 2021). Therefore, it is imperative that future research transitions from fragmented discoveries to systematic integration by constructing comprehensive pathophysiological models, with a particular focus on emerging fields such as immunometabolism, the gut-brain axis, and cerebral microcirculation. The aim of this approach is to increase the accuracy of the mechanism elucidation and to establish the basis of a personalized intervention.

In the process of seeking intervention measures, natural compounds show great potential due to their multi-target effects and relatively low toxicity. Nevertheless, their route towards clinical translation is no less perilous. The difficulties are mainly due to the differences in the species. The dosages and routes of administration applied in animal experimentation to verify drug efficacy may vary considerably with regard to bioavailability in human beings. Absorption, distribution, and metabolism of natural compounds in the human body have their peculiarities and can lead to either inadequate or excessively high effective concentrations at targets in clinical practice (Lu and Xiao-Qing, 2019). Pharmacokinetic studies show that the bioavailability of oral quercetin is only 5.3%, of which about 93.3% is metabolized in the gut (Jing et al., 2023). Its poor water solubility and short half-life further reduce its bioavailability (Eity et al., 2024). Even well-known compounds, e.g., resveratrol, that have been documented to exhibit good bioavailability at the animal level are not well-known in the pharmacokinetic behavior in certain population groups, e.g., the elderly and children. In addition, the uncertainty of dosage safety, the complexity of natural compound metabolites, the lack of unified quality control standards, and the low blood-brain barrier permeability. Although there are currently a large number of *in vitro*, *in vivo*, and clinical studies showing that curcumin contains anti-inflammatory, antioxidant, and anti-viral properties, its relatively large structure and hydrophobic nature lead to poor blood-brain barrier permeability (Godse et al., 2023). The above limiting factors have severely affected the stability and repeatability of efficacy, posing substantial barriers to translation.

### 6.4 Nano-delivery system

In response to the above shortcomings, future studies must emerge in a more precise and integrated direction. Nano-delivery systems are regarded as potential solutions, the innovative application of nanotechnology formulations significantly enhances the brain-targeting delivery efficiency and bioavailability of natural compounds, providing critical support for clinical translation. Polylactic-co-glycolic acid-polyethylene glycol (PLGA-PEG) nanoparticles can effectively penetrate the damaged blood-brain barrier and achieve uniform diffusion in the brain parenchyma; erythrocyte membrane-coated PLGA particles loaded with curcumin exhibit stable sustained-release characteristics, and their surface-modified T807 molecules, by enhancing the binding ability to hyperphosphorylated tau protein, synergistically block key pathological pathways related to Alzheimer’s disease. In a comparison of formulation performance, after intranasal administration, the area under the curve of curcumin complexes encapsulated with hydroxypropyl-β-cyclodextrin-encapsulated curcumin complexes in both brain tissue and plasma was significantly superior to that of curcumin-encapsulated chitosan-coated PLGA nanoparticles. Furthermore, *in vitro* experiments demonstrated a higher cellular uptake rate and superior antioxidant and anti-inflammatory effects. Moreover, curcumin loaded lipid-core nano-capsules effectively alleviated behavioral and neurochemical abnormalities in an Aβ1-42-induced AD model, whereas nanostructured lipid carriers improved memory function by modulating oxidative stress parameters in the hippocampal tissue, such as the ADP/ATP ratio and reactive oxygen species generation (Shabbir et al., 2020).

Intranasal administration, as a non-invasive delivery pathway for the central nervous system, further optimizes the therapeutic window of natural compounds when combined with nanocarriers. Nanocarriers loaded with curcumin delivered intranasally can significantly enhance its bioavailability and blood-brain barrier permeability. Meanwhile, carrier systems such as liposomes, micelles, and inorganic nanoparticles have demonstrated their ability to cross the blood-brain barrier in preclinical studies, and the development of novel delivery platforms, such as thermosensitive hydrogels, further strengthens the translational potential of intranasal administration in the treatment of central nervous system diseases, providing empirical evidence for the precise implementation of a dual intravenous/intranasal delivery strategy (Godse et al., 2023).

### 6.5 Personalised medicine and combination therapies

An important current trend is the deepening of the concept of personalized medicine. Given the significant differences in individual responses to anesthetics and susceptibility to cognitive dysfunction, precision anesthesia strategies based on individual characteristics such as genetics and metabolism are key to reducing risks. In this context, natural compounds may become perfect synergistic therapeutic companions. Within this framework, natural compounds may serve as ideal synergistic therapeutic partners. In particular, it has been shown that quercetin may suppress neuroinflammation and enhance cognitive performance by increasing the expression of miR-138–5p in animal subjects, and the expression level of its target LCN2 can depend on personal genetic predispositions. This further implies that the types and doses of natural compounds as adjuncts might be modified in the future based on the molecular biological properties of the patients and this may enable a level of accuracy never observed before in terms of neuroprotection.

The other important benefit of natural compounds is their multi-mechanistic and multi-targeted action properties, which give the room to design combination therapy approaches. Using quercetin as an example, not only does it regulate via the miR-138–5p/LCN2 axis, but also may be able to affect the downstream Janus kinase 2 (JAK2)/signal transducer and activator of transcription three protein (STAT3) signaling pathway. This multi-mechanistic intervention approach motivates us to actively filter natural compounds that target various pathophysiological pathways, including neuroinflammation, oxidative stress, and synaptic dysfunction and combine them into compound preparations. It is anticipated to overcome the drawbacks of single-drug therapy through the synergistic control of several targets and provide stronger and lasting therapeutic effects (Lou et al., 2025).

Scientifically sound clinical translational research is a crucial route to take to be able to translate the potential of the laboratory into bedside benefits. It is important to formulate clinical preventive measures founded on the explicit molecular processes exposed by fundamental research. As an example, the explanation of the “miR-138–5p/LCN2 axis” indicates that biomarkers of this pathway may be chosen as indicators of efficacy in the conduct of clinical trials, thus developing more specific intervention programs. Simultaneously, the identification of molecular markers of treatment response is extremely important in the development of personalized treatment. It has been demonstrated that silencing miR-138–5p artificially can fully eliminate the protective effect of quercetin, which is a significant indication of the prominent position of the molecule in the response to the treatment. In the future, by promptly measuring the expression of such molecular markers in patients prior to surgery, clinicians can determine the most probable beneficiary groups of the particular natural compound interventions, indeed realizing “individualized treatment” and optimizing clinical response.

Finally, the key to this problem of anesthetic-induced cognitive dysfunction is the presence of safe and effective medications. Presently, natural compounds such as quercetin are also being used as potential drug substitutes offering us with new promising strategic options. Its obvious cognitive protective properties in the animal models and the control of the “miR-138–5p/LCN2 axis” not only prove its developmental utility, but also unveil novel drug action targets. Future research must also examine its direct action targets, long-term safety and optimal delivery formulations to ensure a faster translation of basic research to clinical practice.

Overall, we can unmistakably observe that the breakthroughs of the future rely on the tight collaboration between the depth of fundamental research and the breadth of clinical needs. Through the application of the ideas and methodologies of precision medicine, we will move the field beyond observational phenomena to mechanistic analysis and further beyond mechanistic analysis to effective and personalized clinical interventions, which will, ultimately, lead to the primary objective of neural safety of patients in the perioperative period.

## 7 Conclusion

Cognitive dysfunction induced by anesthesia is a complex neurocognitive disorder and is characterized by a variety of pathological processes, such as neuroinflammation, oxidative stress, neuronal apoptosis, and neuronal plasticity damage. Neuroinflammation impairs neural functionality by stimulating pro-inflammatory cytokines and other signaling pathways (TLR4/NF-κβ and NLRP3 inflammasome), and oxidative stress causes cell damage and apoptosis by overproduction of ROS and imbalance between the antioxidant defense system; simultaneously, the imbalance of neurotransmitters and polarization of microglia can increase the severity of the inflammatory response, impair neuroprotection and repair capabilities, and affect cognitive functional recovery. MiRNA and epigenetic regulation significantly influence the regulation of the above pathological processes and immune inflammatory reactions and become a central point of entry into the investigation of the mechanisms of cognitive dysfunction.

According to this pathophysiological framework, natural compounds have shown significant potential for intervention in anesthesia-induced cognitive dysfunction due to their multi-target and multi-mechanism regulatory abilities. These compounds comprise several types of structures, including flavonoids, saponins, and triterpenes. The mechanism concerned includes primarily the inhibition of pro-inflammatory cytokine release, the activation of the antioxidant signaling pathway, the balancing of expression of apoptotic proteins, the repair of neuronal plasticity, and the regulation of microRNA expression, thus obtaining a multidimensional comprehensive intervention of cognitive dysfunction. Certain natural compounds further have the ability to control microglial cell polarization, induce autophagy, and epigenetic changes, which further emphasize the complexity of their pathological regulation processes.

Nevertheless, the existing studies are not without limitations. Animal models and human clinical conditions are heterogeneous, which poses challenges in the translation of basic mechanisms and clinical pathology. In addition, direct clinical translation is also limited by several factors, including compound bioavailability, pharmacokinetics, and dose safety. In the future, there is an urgent need to establish precise and comprehensive pathological models of cognitive dysfunction, and to leverage multi-omics technologies and single-cell transcriptomics to advance mechanistic research and structural optimization of natural compounds. Flexibly utilize nano-formulations to assist drug delivery and expand personalized treatment plans, in order to achieve the synergistic application of natural compounds and precision anesthesia techniques.

Moreover, the clinical trial design must be informed by the concept of multidimensional assessment of animal studies that combine cognitive behavioral measures, organizational morphology, molecular biology measures, and pathway mechanism validation, thus increasing the scientific rigor and clinical utility of intervention effects. In general, natural compounds have wide research opportunities and are likely to be applied to the prevention and treatment of anesthetic-induced cognitive dysfunction, yet they require more rigorous mechanistic investigations and clinical trials to advance them into scientific translation and clinical practice.

## Glossary

AD: Alzheimer’s disease
Akt: Protein Kinase B
AMPK: AMP Activated Protein Kinases
Arg-1: Arginase-1
ASC: Apoptosis-Associated Speckle-Like Protein
BDNF: Brain-Derived Neurotrophic Factor
Bad: Bcl2-Associated Agonist of Cell Death Protein
Bax: Bcl2-Associated X Protein
CA1: Cornu Ammonis 1 Area
CA3: Cornu Ammonis 3 Area
CD206: Clusters of Differentiation 206
CD86: Clusters of Differentiation 86
ERK1/2: Extracellular-Signal-Regulated Kinase 1/2
GSK-3β: Glycogen Synthasekinase-3β
IL-1β: Interleukin-1beta
IL-6: Interleukin-6
IL-18: Interleukin-18
Inos: Inducible Nitric Oxide Synthase
JAK2: Janus kinase 2
JNK: c-Jun N-terminal Kinase
LCN2: Lipocalin 2
MAPK: Mitogen-Activated Protein Kinases
mTOR: Mammalian Target of Rapamycin
NF-кβ: Nuclear Factor Kappa β
NLRP3: NOD-Like Receptor 3
PARP: Poly-Adenosine Diphosphate-Ribose Polymerase
P-CaMKII: Phospho-Thr286-Calcium-Calmodulin Dependent Protein Kinase II
p-CREB: Phospho-Ser133-CAMP-Dependent Response Element Binding Protein
PI3K: Phosphoinositide 3-Kinase
PLGA-PEG: Polylactic-co-glycolic acid-polyethylene glycol
PSD-95: Postsynaptic Density Protein-95
ROS: Reactive Oxygen Species
SOCS3: Suppressor of Cytokine Signaling 3
SOD: Superoxide Dismutase
STAT3: Signal Transducer and Activator of Transcription 3 Protein
TLR4: Toll-Like Receptor 4
TNF-α: Tumor Necrosis Factor Alpha
XIAP: X-linked Inhibitor of Apoptosis Protein
α7-nAChR: Alpha7-Nicotinic Acetylcholine Receptors
